# Supplementary material for: Myopathy reversion in mice after restauration of mitochondrial complex I
Source: EMBO Mol Med. 2020 Jan 9;12(2):e10674. doi: 10.15252/emmm.201910674 (PMC7005622; doi:10.15252/emmm.201910674)
Supplement: Supplementary file 1 — Appendix [file EMMM-12-e10674-s001.pdf]

Appendix

Myopathy reversion in mice after restauration of mitochondrial complex I

Claudia V. Pereira, Susana Peralta, Tania Arguello , Sandra R. Bacman, Francisca Diaz and Carlos T. Moraes

Appendix Supplementary Figures

Fig. S1. Creation of conditional skeletal muscle NDUFS3 smKO mouse model. Supplementary

Fig. S2. Phenotypic characterization of mouse muscle Ndufs3 smKO.

Fig. S3. Lack of NDUFS3 in the skeletal muscle induced progressive muscle wasting and mitochondrial proliferation.

Fig. S4. Gene replacement at days 15-18 prevented the biochemical phenotype of Ndufs3 smKO.

Fig. S5. rAAV9-derived expression and tissue distribution in young-injected mice.

Fig. S6. rAAV9-derived expression and tissue distribution in adult-injected mice.

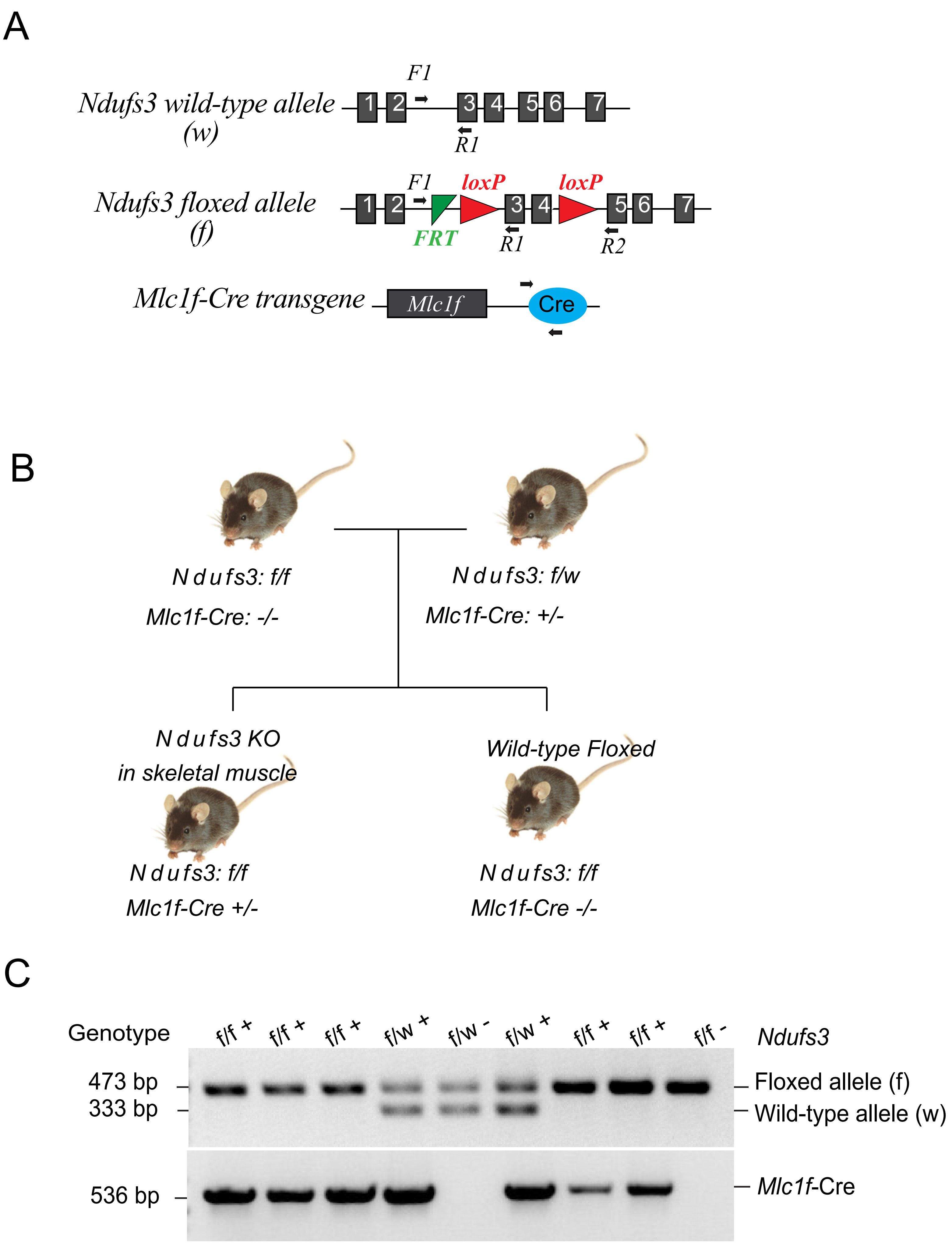

Appendix Fig. S1. Creation of conditional skeletal muscle *Ndufs3* smKO mouse model. (A) Targeting strate-gy for the conditional disruption of *Ndufs3* gene in skeletal muscle. Schematic representation of the *Mus musculus Ndufs3* gene, the *Ndufs3* targeted allele (floxed), and the cre-enzyme is expressed under the myosin light chain pro-moter 1 (*Mlc1f*) specific for skeletal muscle. Black arrows show position of forward (F1) and reversed (R1, R2) primers used for genotyping. **(B)** To generate the *Ndufs3* skeletal muscle-specific conditional knockout, males homozygous for floxed *Ndufs3* (*Ndufs3f/f*) were mated to female mice with the genotype: *Ndufs3(+/f)*, *Mlc1f-Cre(+/-)*. **(C)** Example of genotyping analyses by PCR for *Ndufs3* and *Mlc1f-Cre*.

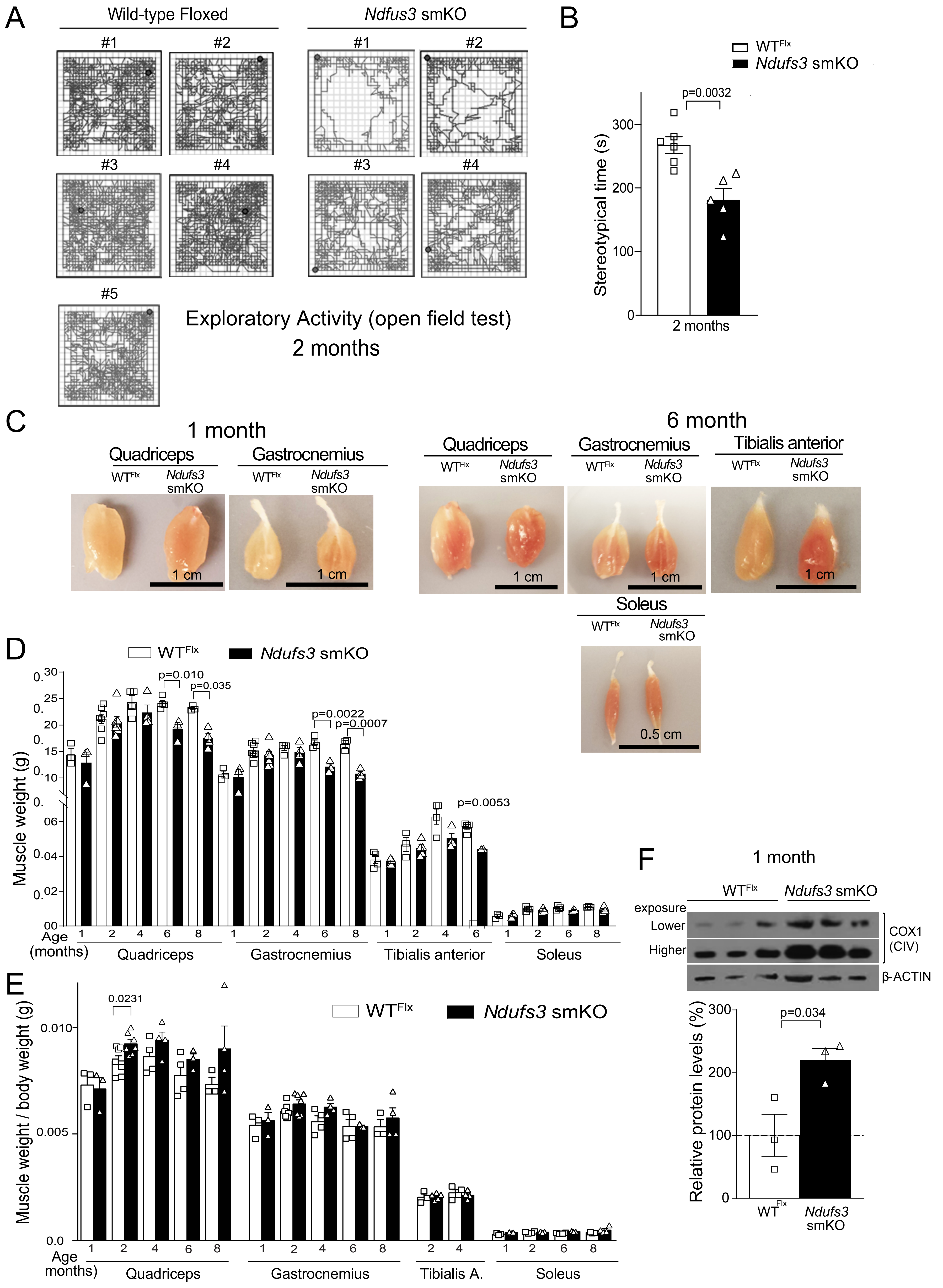

Appendix Fig. S2. Phenotypic characterization of mouse muscle Ndufs3 smKO. (A) Exploratory activity of skeletal muscle specific Ndufs3 smKO and WTFlx mice. Images representing the path traveled by 2-month-old mice during 30 minutes in the Open Field. (B) Stereotypical time during the Open Field test in WTFlx and Ndufs3 smKO groups. Bars represent means ± standard error (SEM) test; (n=5 for WTFlx and n=4 for smKO mice groups). (C) Representative images of skeletal muscles from Ndufs3-KO and WTFlx males at 1 and 6 months of age. Glycolyticmuscles, such as Quadriceps, Gastrocnemius and Tibialis anterior were redder in the smKO. By contrast, no difference in color was found in the oxidative muscle soleus. (D) Absolute muscle weight from Ndufs3-smKO mice (black bar) and age matched WTFlx mice (white bar) at 1, 2, 4, 6 and 8 months (n=3-10). Bars represent means ± standard error (SEM). P values were calculated by Student's t test, n.s.- not significant. (E) Relative muscle weight to total weight in the same samples as panel D. (F) Mitochondrial COX1 protein expression was determined by western-blot in quadriceps homogenates from 1-month-old WTFlx and Ndufs3 smKO mice. Increased levels of COX1 were observed in smKO mice samples. P values were calculated by Student's t test.

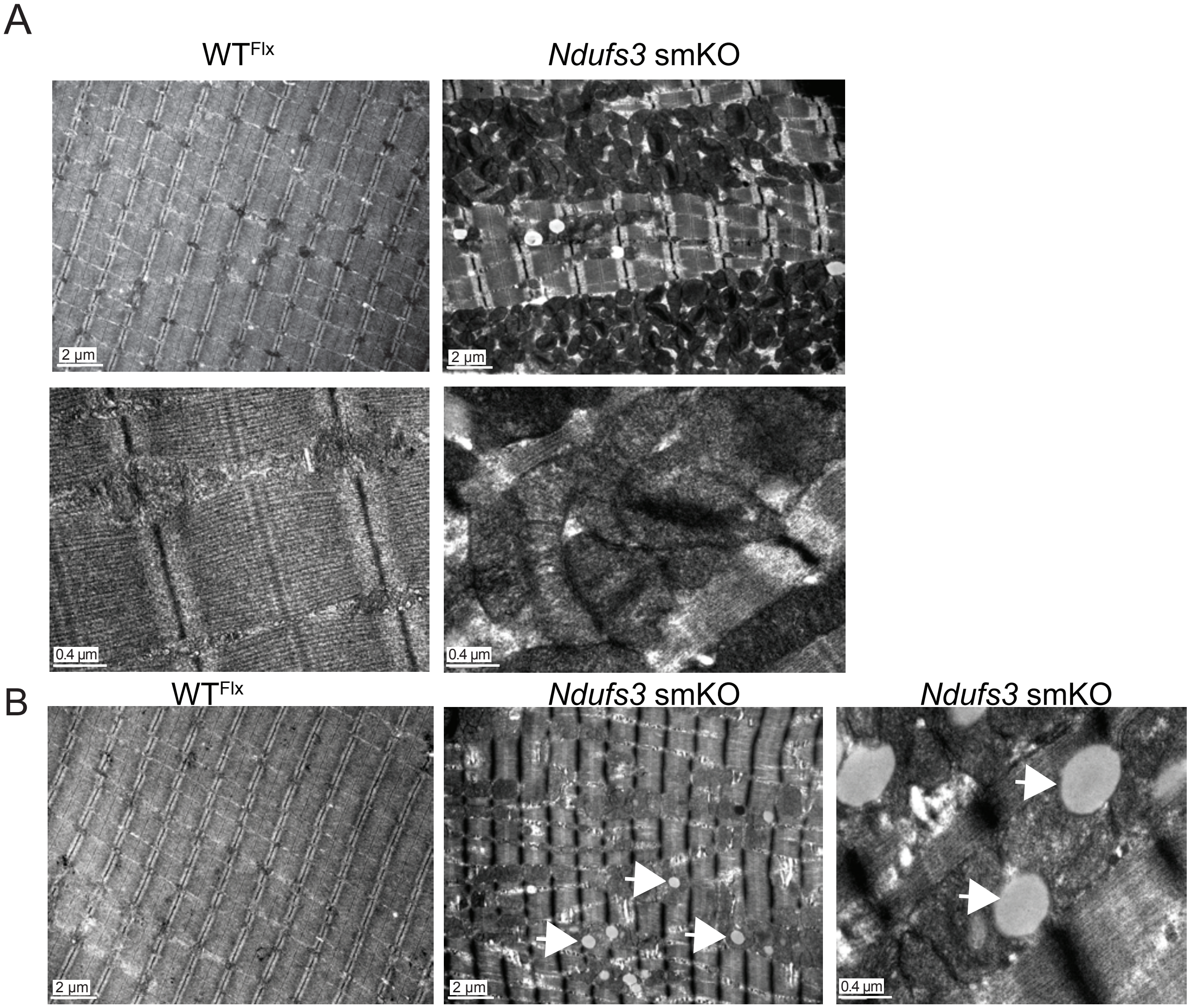

Appendix Fig. S3. Lack of NDUFS3 in the skeletal muscle induced progressive muscle wasting and mitochondrial proliferation. (A) Representative electron micrograph of quadriceps from WT^{Flx} and *Ndufs3* smKO mice at 8 months old. In the smKO mice mitochondria appeared clustered, with condensed cristae and apparent increased number compared to WT^{Flx}. (**B)** Lipid inclusions were observed in the mitochondria of the *Ndufs3* smKO mice, as indicated with white arrow heads.

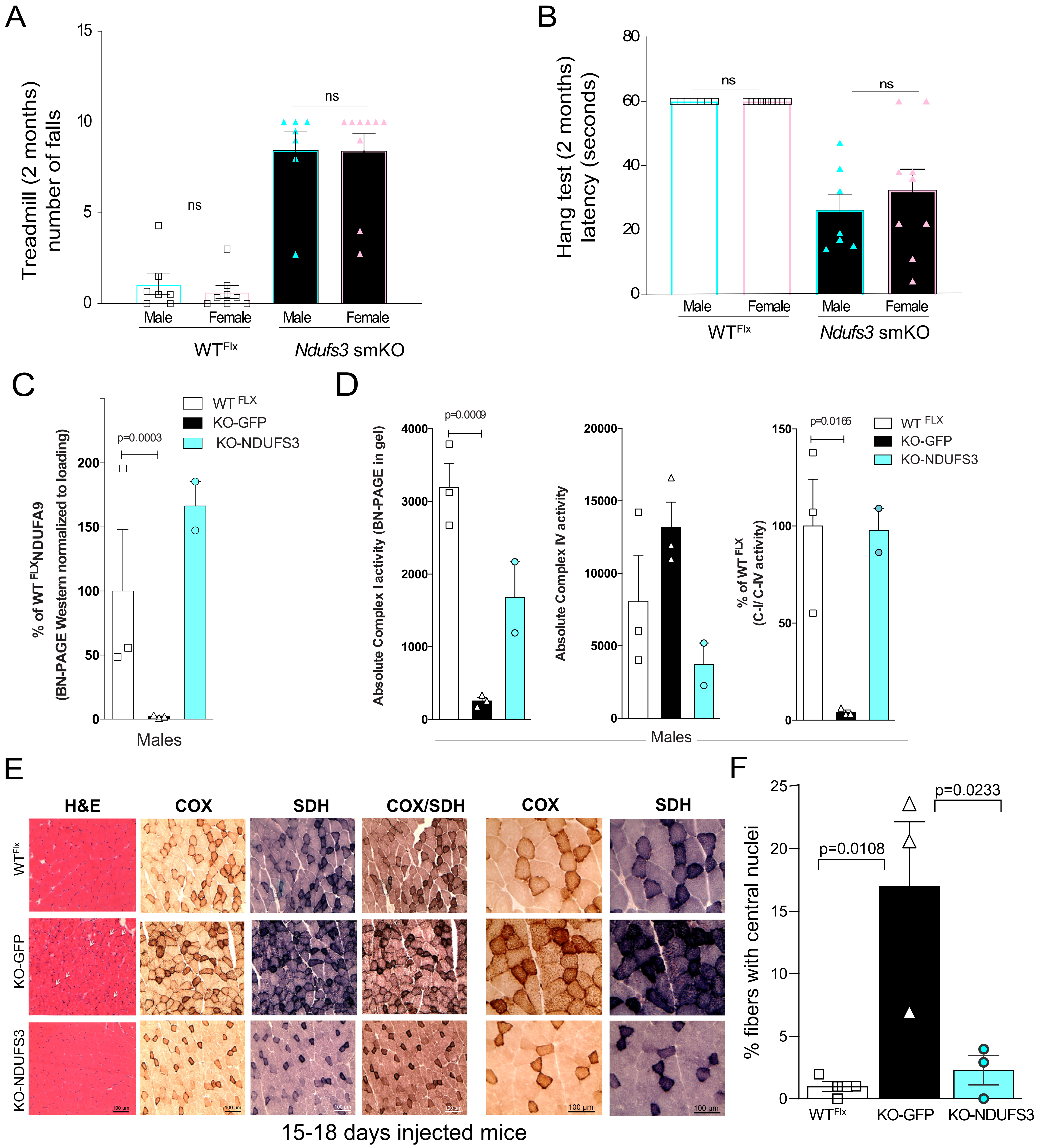

Appendix Fig. S4. Motor skills were reduced in 2 months old Ndufs3 smKO mice and gene replacement at days 15-18 prevented the biochemical phenotype of Ndufs3 smKO. (A) Treadmill performance was significantly reduced in 2 months old Ndufs3 smKO mice, both females and males, when compared to the WTFlx littermates. (B) Hang test measured as the latency to fall was significantly reduced in 2 months old Ndufs3 smKO mice, both females and males, when compared to the WTFlx littermates. (C) Quantification of CI from BN-PAGE showed in Figure 4 panel C, males. (D) Quantification of BN-PAGE in-gel activity showed in Figure 4 panel F. (E) H&E staining and COX/SDH activity staining in muscle sections of KO-NDUFS3 were similar to WTFlx mice. (F) The percentage of central nuclei in skeletal muscle fibers was determined in quadriceps sections stained with H&E, n=3 mice, per group. The results showed no differences between the WTFlx and KO-NDUFS3, whereas KO-GFP samples had increased number of fibers with central nuclei. Error bars represent ±SEM. Statistical analysis was performed by One-way ANOVA followed by Bonferroni post-test.

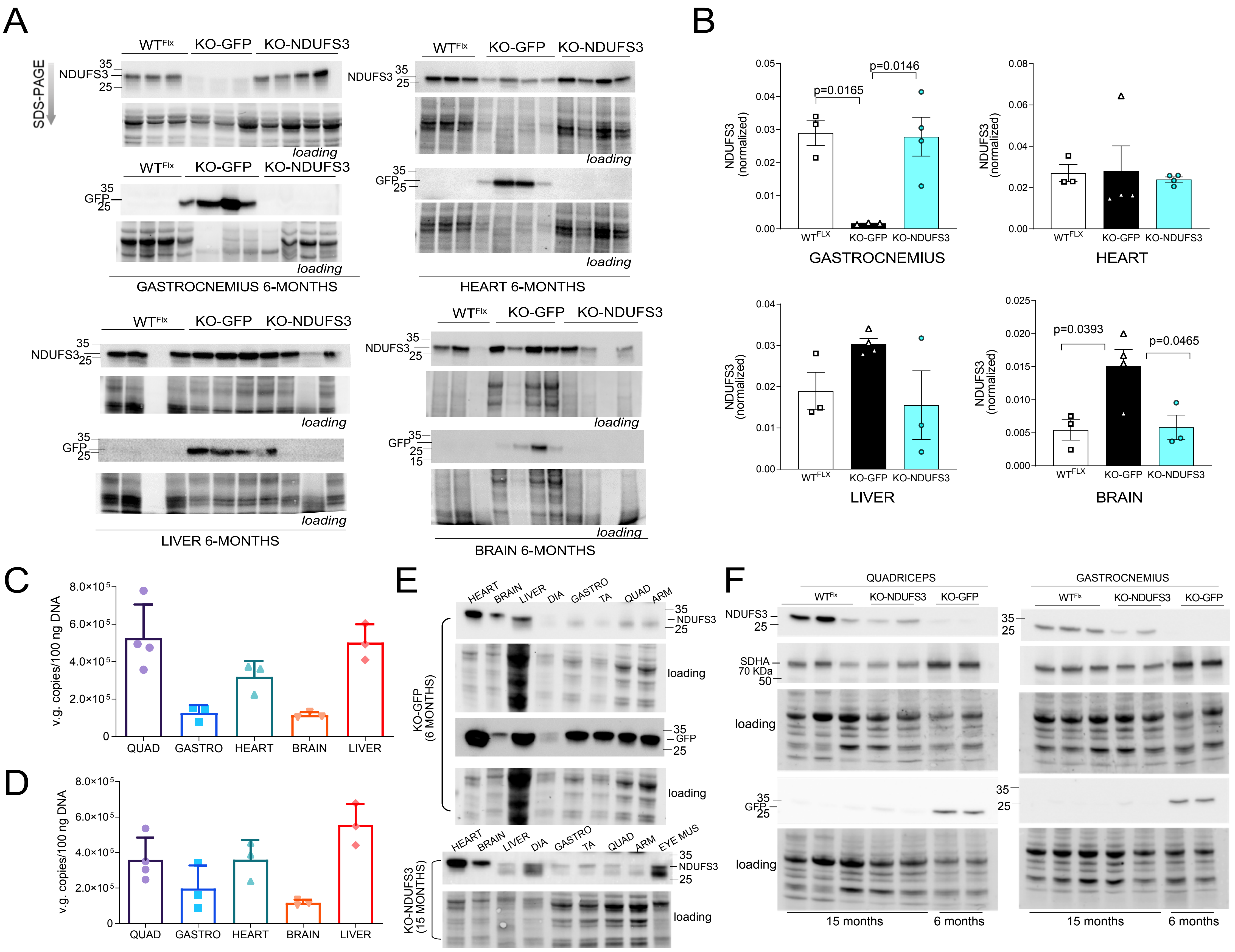

Appendix Fig. S5. AAV9-derived expression in young-injected mice tissues. (A) Gastrocnemius and other tissues such as the heart, liver and brain were analyzed for NDUFS3 and GFP protein expression by western blot. GFP expression was observed in all tissues analyzed. NDUFS3 expression was restored in gastrocnemius of KO-NDUFS3 mice samples. (B) Quantification of western blots shown in (A). (C) Quantitative PCR determination of viral genome (vg) copies per 100 ng of DNA from KO-NDUFS3 mice tissues normal-ized by non-injected mice samples. The probe used targeted the AAV9-NDUFS3 plasmid in the CMV promoter region. (D) Sames as (C) with a probe targeting the bGH region of the original vector. (E) Different skeletal muscle and other tissues were analyzed for NDUFS3 and GFP protein expression by western blot. GFP expression was observed in all the tissues analyzed. DIA-diaphragm; GASTRO-gastrocnemius, TA-tibialis anterior; QUAD-quadriceps, ARM-triceps and other arm muscles, EYE MUS-eye muscles. (F) Comparison of WT^{Flx}, KO-NDUFS3 and KO-GFP protein expression (NDUFS3, SDHA and GFP) in quadriceps and gastrocnemius samples of P15-18 injected mice. For (E) and (F) WT^{Flx} and KO-NDUFS3 samples were collected at 15 months whereas KO-GFP samples were collected at 6 months.

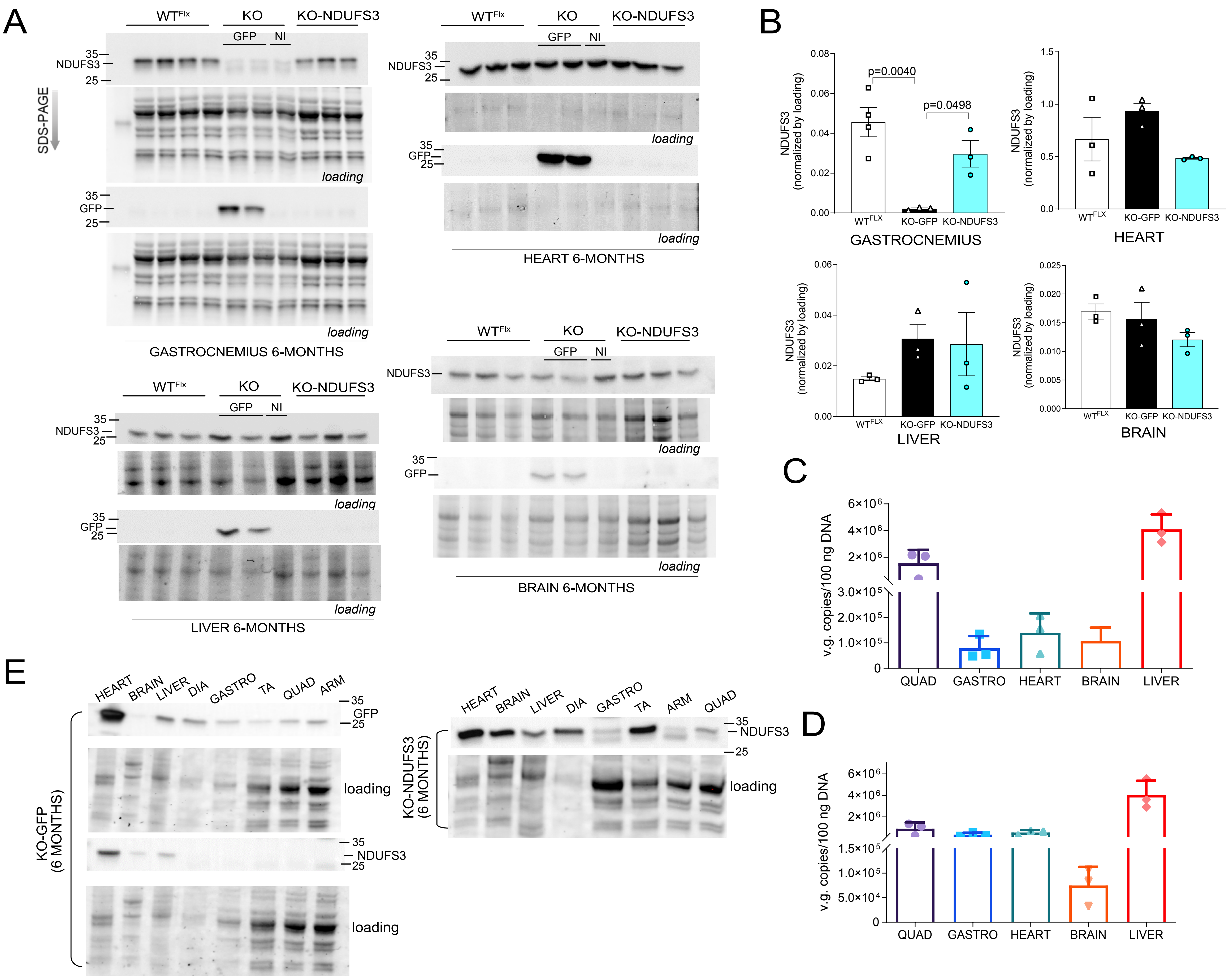

Appendix Fig. S6. AAV9-derived expression in adult-injected mice. (A) Western blots of gastrocnemius, heart, liver and brain samples. NDUFS3 expression was rescued in KO-NDUFS3 gastrocnemius samples. GFP expression was observed in all tissues analyzed. (B) Western-blot quantification of (A). (C) Viral genome copies determination by qPCR showed expression of the AAV9-NDUFS3 in different tissues, besides the skeletal muscles analyzed. The probe used was targeted to the CMV promoter region of the AAV9-NDUFS3 plasmid. (D) Same as (C) but the probe used was targeted to the bGH region. (E) Comparison of WTFlx, KO-NDUFS3 and KO-GFP by western blot. NDUFS3 and GFP were expressed in gastrocnemius samples of 2-month-old injected mice. All samples were collected at 6 months.
